# 25, 50 and 75 years ago

**DOI:** 10.1111/ans.17929

**Published:** 2022-08-11

**Authors:** Julian A. Smith

**Affiliations:** ^1^ Department of Surgery Monash University Melbourne Victoria Australia

## 25 years ago

**Isbister WH. Colorectal surgery in the elderly: an audit of surgery in octogenarians. *ANZ J Surg 1997;67:557–561***

Morbidity and mortality rates are higher in elderly compared to younger patients undergoing colorectal cancer surgery. This study was undertaken to see whether this finding applied to all colorectal surgery in the elderly and if so to try to identify the determining factors. All patients undergoing colorectal surgery between 1975 and 1990 were entered into a computerized database. Patients were divided into two groups, those less than 80 years (<80) and those 80 years and more (80+), and compared with regard to the type of surgery performed, the patient's race, the seniority of the surgeon, the patient's disease, the operation performed and the postoperative morbidity and mortality. In addition, patients undergoing major resectional surgery and patients undergoing colorectal cancer surgery were compared separately. Of 2011 admissions, 88 were for patients of 80+. The male to female admission rate was 1: 0.79 in the <80 group and I: 1.25 in the 80+ group. More surgical procedures were performed by consultants in older patients. More emergency admissions were for 80+ patients. Rectal, sigmoid and right colonic pathology was more common in the elderly. Very few elderly patients were admitted with minor anorectal problems. Rectal prolapse and colorectal cancer were the commonest causes for admission in octogenarians. There were more pulmonary and cardiovascular postoperative complications in 80+ patients. Urinary tract infections were also more common. The postoperative mortality rate was higher in older patients (7.9 versus 1.4%). Four hundred and sixty‐two patients underwent major resectional surgery and 45 were 80+. Surgery for diverticular disease was more frequent in younger patients (13.4 VS 2.2%) and cancer surgery in older patients (93.3 versus 70.5%). The postoperative mortality rate was higher in the elderly (11.1 versus 3.6%). Three hundred and thirty‐six major resections were for cancer and 42 were 80+. Emergency surgery was performed more commonly in the older group (38.1 versus 14.9%). The rate of advanced disease seemed to be similar in both groups. The postoperative death rate was higher in the elderly (11.9 versus 3.4%). Elderly patients were more likely to die from cardiopulmonary problems after surgical interventions than either from their primary disease or from the surgery undertaken for it. Good postoperative cardiopulmonary support should thus be provided for all such patients.

**Stephens FO. Newer approaches to regional cancer therapy through tumour immunology: is there a ‘breakthrough’? *ANZ J Surg 1997;67:2–4*.**

Eagerly awaited ‘breakthroughs’ in immunological treatment have in the past been disappointingly unsuccessful in changing the outlook for most patients with otherwise incurable cancers. Many hopeful agents have been studied in therapeutic trials but each in turn has proven to be largely disappointing. One of the latest products of immunological research, tumour necrosis factor (TNF) was found to be too toxic for systemic use but has been found to be highly effective in improving the results of treatment of melanoma when used in a closed‐circuit perfusion system in combination with another chemotherapeutic agent. In the past the use of closed‐circuit perfusion has been confined to limbs, but techniques have recently been developed to apply closed‐circuit perfusion to liver, pelvic organs, and some abdominal regions including pancreas. The potential for studies of TNF in combination with chemotherapy in closed‐circuit perfusion treatment of otherwise resistant cancers in these organs and tissue regions has been greatly expanded. In many cancer treatment centres in the past there has been a reluctance to use and to acknowledge the benefits of regional delivery of anti‐cancer chemotherapy. The need for these techniques in the safe and effective use of TNF has further confirmed the importance of these methods in comprehensive cancer treatment centres, and the need for further studies and better understanding of the use of regional and closed‐circuit perfusion methods.

## 50 years ago

**Pheils MT. Vesico‐colic fistula due to diverticulitis. *ANZ J Surg 1972;41:237–240***

**Fig. 1:**
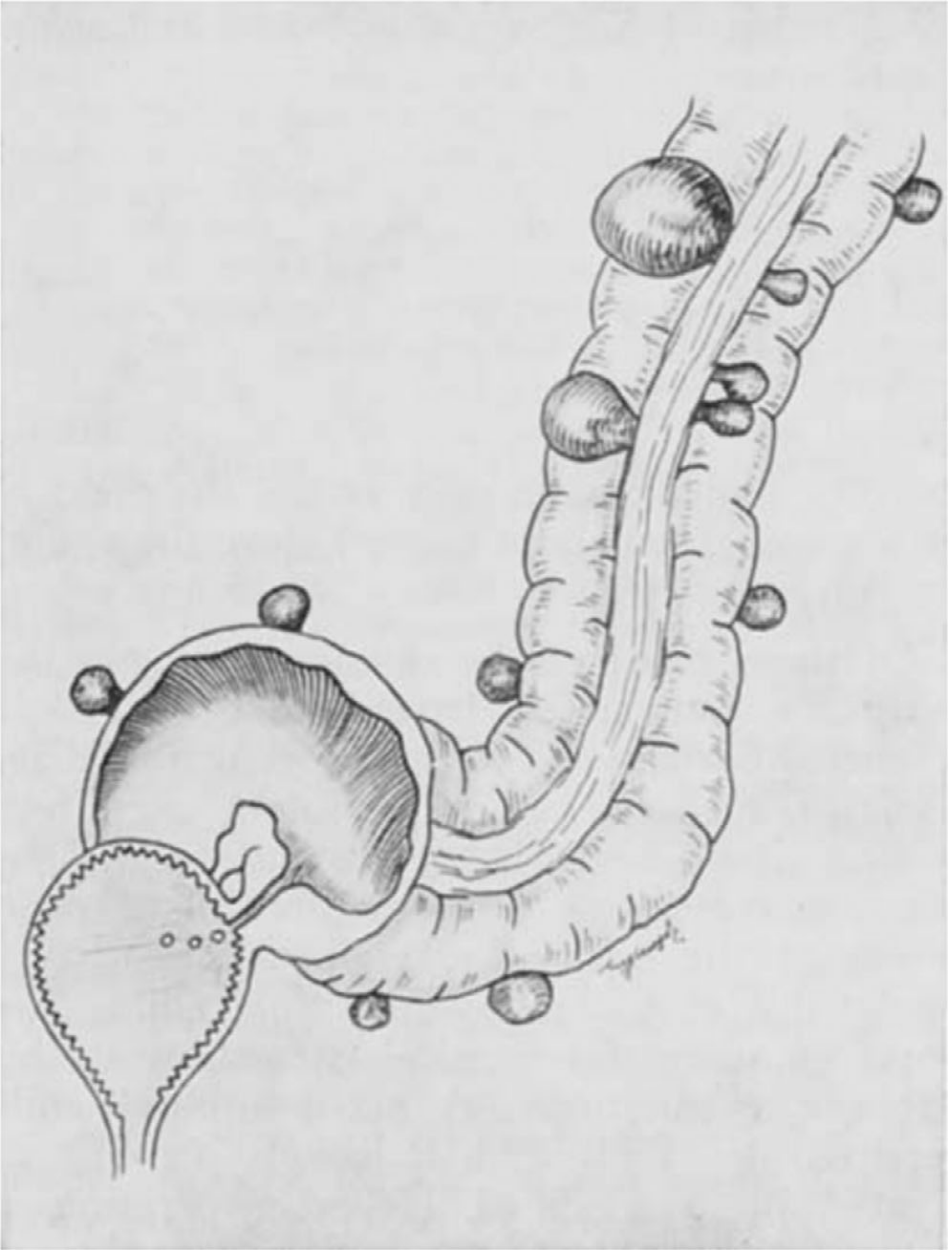
Established vesico‐colic fistula.

**Fig. 2:**
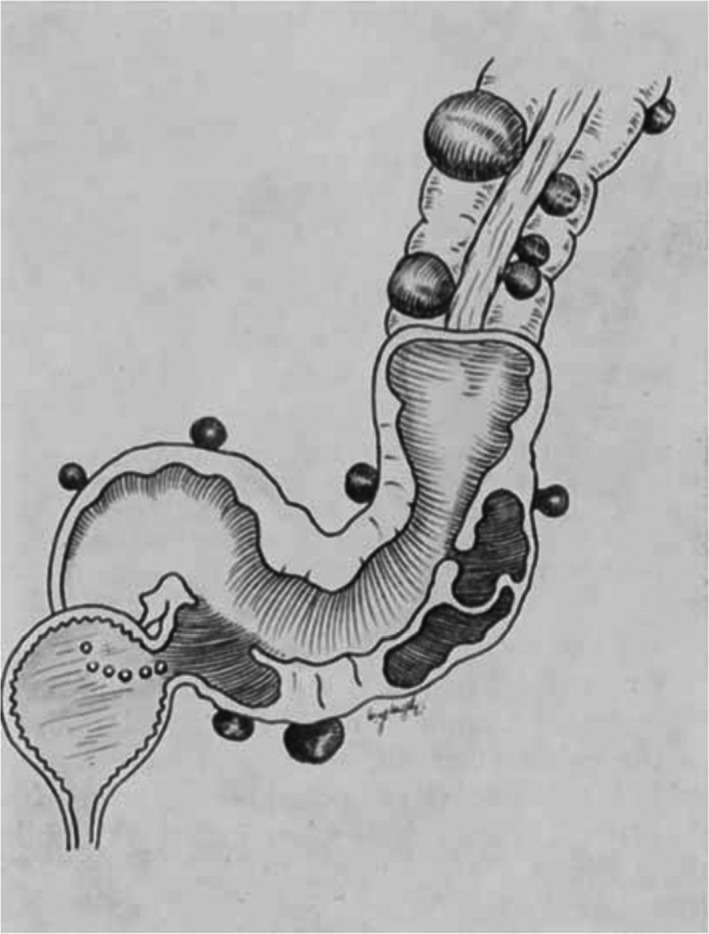
Pericolic abscess rupturing into the bladder.

**Fig. 3:**
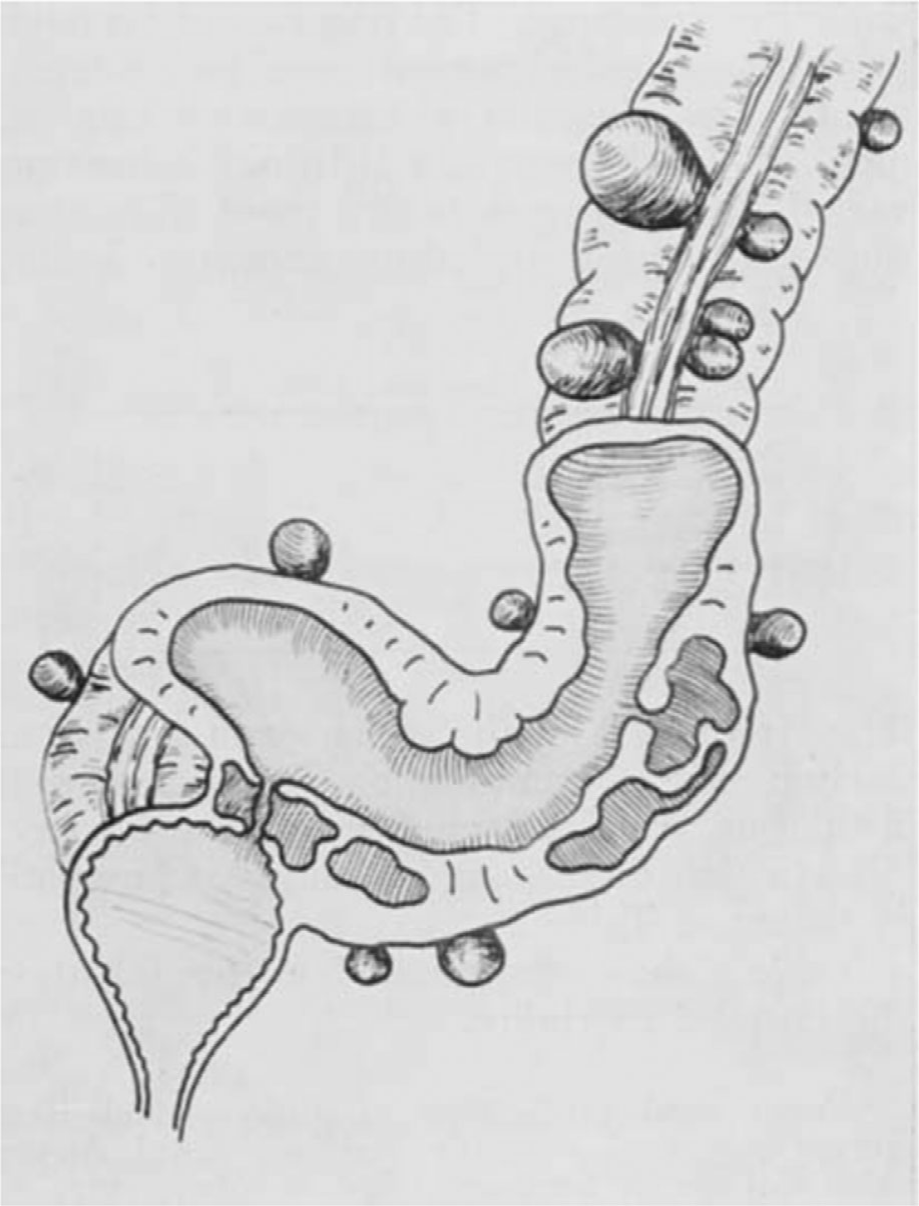
Pericolic abscess and phlegmon adherent to the bladder.

Fifty‐five patients treated for an established vesico‐colic fistula (Fig. 1) due to diverticulitis were studied. The condition occurs most frequently in the elderly male, pneumaturia is the most frequent symptom, and it is only possible to demonstrate the fistula by cystoscopy or barium enema examination in about one‐third of the cases. A suggested classification of the cases into three groups has been made, on the basis of the presence of:

1. Rupture of a pericolic abscess into the bladder (Fig. 2)
2. Diverticulitis with a pericolic abscess or phlegmon attached to the bladder (Fig. 3); or
3. Rupture of one diverticulum into the bladder.

A staged operation is recommended for Groups 1 and 2, and a one‐stage resection and anastomosis for Group 3.

**Stuart M. Radical resection of rectal cancer. *ANZ J Surg 1972;42:151–155*.**

Radical resection of the rectum is a widely practised treatment for rectal carcinoma whether for cure or palliation. However, this approach is attended by a significant postoperative mortality and morbidity, with a five‐year survival rate rarely exceeding 50%. The same experience has been found at the Prince Henry and Prince of Wales Hospitals, which are major teaching hospitals of the University of New South Wales. In view of these findings it is suggested that no longer should the diagnosis of carcinoma of the rectum mean automatically that a radical resection of the rectum must be carried out. Instead, a more selective approach is outlined, with lesser procedures in selected cases. This more selective approach is in keeping with current trends in the management of such conditions as breast carcinoma and malignant melanoma. In any given patient with a rectal carcinoma, due consideration must be given to its size, site, shape, histological features and potential curability. Only then can a decision be made as to whether radical resection is indicated or whether one or more of the lesser procedures discussed here will in fact benefit the patient more.

## 75 years ago

**Starr KW. The mechanical fixation of fractures of the shafts of the long bones. *ANZ J Surg 1947;16:248–252***

In some 500 fractures encountered in military patients only 122 have *been* mechanically fixed, so that a conservative attitude has been my practice. The tendency is, however, to employ mechanical fixation when displaced fractures are unstable, anaemic or septic. My experience tends also to emphasize the importance of employing it as a primary measure and not to regard it as a secondary resource to snatch a brand from the burning. The circumstances which influence me to adopt mechanical fixation are five in number:

1. Displacement increases the area of the fracture site, damages blood supply from the periosteal and endosteal sources, and in weight‐bearing bones predisposes the adjacent joints to traumatic arthritis if not corrected.
2. Instability is an inherent characteristic of certain fractures (spiral tibial and forearm fractures, the lower radial third *et cetera)*. Others are unstable because the fracture immobilization must be disturbed for the treatment of infection of the wound or of associated soft tissue lesions preparatory to grafting or closure, or for operative management of brain, chest or abdominal lesions.
3. Poverty of blood supply characterizes fractures of the intracapsular femoral neck and of the lower tibial and ulnar diaphyses because muscular attachments are absent.
4. Sepsis is a more complex story. Cachexia may appear if sepsis is not controlled, and produces mental depression, high fever, marked secondary anaemia, hemoconcentration and a grave negative nitrogen balance. Locally, dead bone, loose or fixed, may be present and suppuration may be profuse, with loculation or gravitational abscesses.
5. Interposition of soft tissues is possible only when displacement is present and may delay union especially in humeral shaft and malleolar fractures.

